# Health policy and integrated mental health care in the SADC region: strategic clarification using the Rainbow Model

**DOI:** 10.1186/s13033-016-0081-7

**Published:** 2016-07-22

**Authors:** André Janse van Rensburg, Pieter Fourie

**Affiliations:** Health and Demographic Research Unit, Department of Sociology, Ghent University, Korte Meer 5, 9000 Ghent, Belgium; Department of Political Science, Stellenbosch University, Corner Merriman and Ryneveld Street, Stellenbosch, 7602 South Africa; Centre for Health Systems Research & Development, University of the Free State, Nelson Mandela Road, Bloemfontein, 9300 South Africa

**Keywords:** Integrated health care, Mental health systems, SADC, Rainbow model, Health policy

## Abstract

**Background:**

Mental illness is a well-known challenge to global development, particularly in low-to-middle income countries. A key health systems response to mental illness is different models of integrated health care, especially popular in the South African Development Community (SADC) region. This complex construct is often not well-defined in health policy, hampering implementation efforts. A key development in this vein has been the Rainbow Model of integrated care, a comprehensive framework and taxonomy of integrated care based on the integrative functions of primary care. The purpose of this study was to explore the nature and strategic forms of integrated mental health care in selected SADC countries, specifically how integrated care is outlined in state-driven policies.

**Methods:**

Health policies from five SADC countries were analysed using the Rainbow Model as framework. Electronic copies of policy documents were transferred into NVivo 10, which aided in the framework analysis on the different types of integrated mental health care promoted in the countries assessed.

**Results:**

Several Rainbow Model components were emphasised. Clinical integration strategies (coordination of person-focused care) such as centrality of client needs, case management and continuity were central considerations, while others such as patient education and client satisfaction were largely lacking. Professional integration (inter-professional partnerships) was mentioned in terms of agreements on interdisciplinary collaboration and performance management, while organisational integration (inter-organisational relationships) emerged under the guise of inter-organisational governance, population needs and interest management. Among others, available resources, population management and stakeholder management fed into system integration strategies (horizontally and vertically integrated systems), while functional integration strategies (financial, management and information system functions) included human resource, information and resource management. Normative integration (a common frame of reference) included collective attitude, sense of urgency, and linking cultures, though aspects such as conflict management, quality features of the informal collaboration, and trust were largely lacking.

**Conclusions:**

Most countries stressed the importance of integrating mental health on primary healthcare level, though an absence of supporting strategies could prove to bar implementation. Inter-service collaboration emerged as a significant goal, though a lack of (especially) normative integration dimensions could prove to be a key omission. Despite the usefulness of the Rainbow Model, it failed to adequately frame regional governance aspects of integration, as the SADC Secretariat could play an important role in coordinating and supporting the development and strengthening of better mental health systems.

**Electronic supplementary material:**

The online version of this article (doi:10.1186/s13033-016-0081-7) contains supplementary material, which is available to authorized users.

## Background

Mental illness is readily recognised as a significant challenge to global development outcomes [1–5]. In 2010, mental, neurological and substance abuse disorders accounted for 258 million disability-adjusted life years (DALYs)—10.4 % of all-cause DALYs—which amounted to an increase of 41 % since 1990 [6]. It is estimated that by 2020 unipolar depression will be second among causes for disability worldwide [7]. The economic costs associated with mental illness are especially significant: the global costs amounted to US$ 2.5 trillion in 2010, and are projected to increase to US$ 6 trillion in 2030 [8]. Spending on mental health care is disproportionate across different regions, with low-to-middle income countries (LMICs) spending US$ 1.53, upper-middle income countries spending US$ 1.96, and high income countries spending US$ 58.73 per capita on mental health in 2013 [9]. A recent economic analysis framed mental illness as a developmental rather than pure public health challenge, suggesting that the total investment required to address depression and anxiety disorders in 36 countries from 2016 to 2030 amounts to US$ 147 billion. However, the returns on investment in this study was calculated to have a benefit to cost ratio of 3.3–5.7 to 1 when considering the value of both economic and health benefits [10]. The study included LMICs in its analysis, where the lack of mental health investment is especially tangible.

Mental illness exerts particular pressure on countries with underdeveloped health systems, which already have to contend with significant challenges associated with poverty, conflict and communicable diseases such as HIV [11–13]. Suicide rates have been suggested to be highest in LMICs, and due to inadequate support systems individuals and their families are disproportionally affected by mental disorders [14]. A major form of health system capital—mental health professionals—are also severely lacking in LMICs: in Africa there are 1.4 mental health workers per 100,000 population compared to the global average of 9 per 100,000 [9]. Despite global efforts towards the strengthening of mental health systems [15, 16], mental health remains on the periphery of the global health agenda [17, 18]. Development assistance for global mental health increased between 2007 and 2013, but remains low—the proportion of the development assistance attributed to mental health is calculated to be less than 1 % of the US$ 133.57 million total amount spent [19]. LMICs especially struggle to attract funding and buckle under chronic underfunding and lack of investment in services [1].

Against the backdrop of these global health governance dynamics, national governments increasingly have to strike an uncomfortable balance between responding to psychiatric need in the population, on the one hand, and producing gain in terms of cost effectiveness, on the other [20, 21]. This tension has led to the adoption of different models of integrated care, an intervention with promising clinical outcomes with possible reduced costs [22–24]. Integrated care is a complex construct [25–27], and while integrated care has emerged as a central feature of mental health system reforms in LMICs [13, 28–30], little attention has been paid to its forms and strategies in policy. The purpose of this article is to explore the nature and strategic forms of integrated mental health care in selected countries in the South African Development Community (SADC) region, specifically how integrated care is outlined in state-driven health policies.

### Conceptualising integrated health care

An integrated care approach is consistently underlined as a strategy to address fragmented and uncoordinated health systems [31], and to increase accessibility to care (especially of disadvantaged communities) [32]. It is well established that integrated care is a multi-layered construct [25–27], and many authors have attempted to pin its meaning down. Integrated care has been used to describe the linking of services or programmes on similar levels of health care (for instance a multidisciplinary, integrated approach to diabetes mellitus management [33]), known as horizontal integration, and to the linking of services or programmes on different health care levels (for instance primary and secondary level integration for the management of serious psychiatric disorders [34]), known as vertical integration [35]. Within this broad categorisation, integrated care has been used to refer to as a patient-centred, demand-driven linking of the health care system with other human service systems on multiple levels to address complex health needs [25, 36, 37]; the consolidation of a range of behavioural, medical and other elements into a single care or service package [26]; the creation of an organisational network providing a coordinated continuum of services to a defined population [38, 39]; the amalgamation of continuity of care, shared care and seamless care [40]; an organising principle that aims to improve care through improved coordination of methods, processes and models in line with the patient’s perspective [41]; and as the collaboration of multiple professionals, organisations and sectors towards coordinated care [42].

It is noteworthy that most conceptualisations of integrated care has been penned in high-income country contexts, and often do not adequately reflect LMIC health system configurations and processes. In this respect the WHO has been a key driver in the introducing of suitable models of integrated mental health care to LMICs. In an influential report, the WHO together with the World Organization of Family Doctors provided guidance on integrating mental health into primary healthcare (PHC) [39]. Given the proliferation of PHC as a foundation of health systems (especially) in LMICs, it made sense to use that as a platform from which to increase mental health care access. Many countries such as Brazil, South Africa and Uganda have in varying degrees introduced initiatives where mental health service capacity is fostered in PHC settings, whether this means training existing health workers or task-shifting related duties to lay health workers. Importantly, such clinical models of integration should be backed by solid national policy frameworks [39]. Eaton and colleagues [28] provide an overview of different models of integrated mental health care that are recommended for and have been adopted by LMICs. These include: task-shifting mental health service provision from psychiatrists and psychologists to nurses and lay health workers; sharing mental health services with other core programmes such as immunisation, chronic conditions and HIV; task sharing to include support from families and community members, integrating mental health indicators within existing health information systems; and stronger collaboration with non-governmental organisations (NGOs). Common elements of integrated care in health policies in African contexts include decentralisation, as well as integrating mental health with general health services, especially at PHC level [7].

Within the multitude of voices, there clearly has been a need to distil the many integrated care types and meanings. Taking into account the integrative functions of primary care, Valentijn and colleagues [43, 44] recently presented the Rainbow Model of Integrated Care, a comprehensive framework and taxonomy of integrated care. Firstly, the authors argue that the primary care principles of first contact care, continuous care, comprehensive care, and coordinated care play a central role in the integration of care. Then, integrated care is structured conceptually along micro, meso and macro dimensions. Macro level integration refers to vertical and horizontal system integration. On the meso level, integration is conceptualised to happen in terms of inter-organisational integration, through market, hierarchy, and network mechanisms, as well as in terms of partnerships between professionals within and between different organisations. Micro-level integration refers to clinical integration, that is, achieving a coherent and coordinated process of health care delivery to individual patients. Linking the macro, meso and micro levels of integration are functional and normative integration. Functional integration refers to “Key support functions and activities…structured around the primary process of service delivery, to coordinate and support accountability and decision-making between organisations and professionals to add overall value to the system.” Normative integration is defined as “The development and maintenance of a common frame of reference (i.e., shared mission, vision, values and culture) between organisations, professional groups and individuals” [43]. These domains of integrated care were subsequently fleshed out in a more comprehensive typology (see Additional file 1). Though still relatively novel, the Rainbow Model has already been used to measure integrated care in the Singapore Regional Health System [45], and its comprehensive underpinnings present a robust framework with which to explore integrated mental health care in national policies.

### National and regional policy and integrated mental health care

Omar and colleagues [46] suggest that “Mental health policies signal a government’s intent to address the mental health needs of its citizens”. In this vein, clarity of conceptualisations in health policy is paramount to the successful establishment of its intentions in implementing strategies, and it helps “to transport the issue from the ideological plane into practice, from a normative approach to a positive one” [47]. Coherent individual as well as communal understanding and sense-making of the components and purpose of complex social interventions are crucial mechanisms in its implementation [48, 49]. Although governing integrated mental health care has been a global challenge, it is especially Sub-Sahara African countries that have struggled to both develop and implement mental health policies [46]. The wide-spread socio-economic inequalities in this particular region not only elevates the pressing need for integrated mental health care, but also contributes to mental illness [50]. Many LMICs and key regional organisations place increased focus on mental disorders [3]. This increased focus is evident in the national strategic mental health care reforms that have taken place to varying degrees in Sub-Saharan African countries during the past decade [7, 12, 29, 30, 46, 51–59].

Regional governing bodies certainly have a part to play in mental health system reforms, the importance of which is underscored during the on-going Ebola crisis in West Africa [60]. The advent of global health governance highlights the interdependence of states and the increasing complexity of illness and disease responses, calling for cooperation among countries on issues that transcend national boundaries [61]. Regional and interstate collaborative governance have been shown to be an effective vehicle with which to address complex health system challenges, as exemplified by the activities of the Union of South American Nations and the WHO’s South East Asian Regional Office [60]. A key regional body in the Sub-Sahara African region is the Southern African Development Community (SADC).^1^ Its Secretariat (seated in Gaborone, Botswana) have not forwarded any tangible policies related to mental health care, and has no dedicated body concerned with health or mental health. Notwithstanding these shortcomings, the SADC Secretariat did produce a Protocol on Health [62] which included a focus on mental health, and have shown promise in its development of cross-border initiatives for malaria and HIV [60].

Many SADC countries are yet to produce dedicated mental health policies. Limited evidence however suggest that in many African countries where mental health policies have been produced, these are often inappropriate, poorly implemented, and not translated into a detailed strategic action plan [46]. In the absence of regional coordination from SADC, it is unclear which types of integrated mental health care are pursued by its members. Clarity in this matter is important, since the effectiveness of mental and neuropsychiatric disorders have been noted to be “largely determined by the health systems in which they are nested” [63]. Geopolitical health system differences (and associated socio-economic inequities) in many ways define the types of integrated mental health care unfolding in countries and regions [64]. For instance, in some countries such as Belgium [65], Canada [66] England and the Netherlands [64] integrated mental health care usually refers to collaborative activities among different, independent service providers. On the other hand, in many African countries integrated mental health care usually refers to the integration of mental health care into general health services, specifically on PHC level [29, 30, 53–59]. These definitions are however highlighted by empirical field studies, and it remains unclear how integrated mental health care unfolds in national policy. Given the pressing need to study health policy and how it frames health systems [67–69], the aim of the study was to explore the scope and focus of integrated mental health care in the SADC region.

## Methods

In order to scrutinise the scope and focus of integrated mental health care in the SADC region, a policy analysis approach was pursued. A key part of policy analysis is to consider the influence of “ideas (arguments and evidence), over health system operations and policy change within them” [69]. National policy documents of five countries were scrutinised: Botswana, Malawi, Namibia, South Africa, and Zambia. Apart from Zimbabwe, these are the only countries with established national mental health policies in the SADC region (attempts to gain access to Zimbabwe policy documents were unsuccessful). Three policy documents were analysed from each country: the national health policy, the national health strategy, and the national mental health policy (see Box 1 for a breakdown). Far from being an exhaustive list of policies, the structuring of mental health service provision is not only contingent on these three types of policies, and many other strategies are potentially important—such as policies related to human resources for health, PHC, chronic illnesses, and so on. This being said, for the purpose of this article a national mental health policy certainly is most important, in addition to a national health policy. Also, strategic plans are important manifestations of national intent in terms of policy change, and so they were included. The SADC Secretariat offers little in terms of mental health-related strategy documents, the closest being its Protocol on Health [62]. However, this particular regional strategy was not included in the formal analysis due to its lack of explicit focus on mental health care. Electronic versions of the documents were imported into NVivo (ver. 10) [70], and sections dealing with integrated care were thematically arranged within the 59 items of the Rainbow Model [44]. NVivo allowed for the systematic analysis of documents, by providing a template within which researchers could thematically arrange integrated mental health care nodes. More specifically, a framework method was followed as described by Gale and colleagues [71]. The thematic arrangement process was checked by and discussed with a researcher who was not part of the study, in order to increase trustworthiness. Both explicit and implicit indications of integrated mental health care were included.

**Box 1:** Policy documents included in the study

Country	Document
Botswana	*National Health Policy: Towards a Healthier Botswana (2011)*
	*Integrated Health Service Plan: A Strategy for Changing the Health Sector For Healthy Botswana 2010*–*2020 (2010)*
	*National Policy on Mental Health (2003)*
Malawi	*To the Year 2020: A Vision for the Health Sector in Malawi (1999)*
	*Malawi Health Sector Strategic Plan 2011*–*2016 (2010)*
	*National Mental Health Policy (2001)*
Namibia	*National Health Policy Framework 2010*–*2020 (2010)*
	*Ministry of Health and Social Services Strategic Plan 2009*–*2013 (2009)*
	*National Policy for Mental Health (2005)*
South Africa	*White Paper for the Transformation of the Health System in South Africa (1997)*
	*Department of Health Strategic Plan 2014/15*–*2018/19 (2014)*
	*National Mental Health Policy Framework and Strategic Plan 2013*-*2020 (2012)*
Zambia	*National Health Policy (2011)*
	*National Health Strategic Plan 2011*–*2015 (2011)*
	*Mental Health Policy (2004)*

## Results

The assessment undertaken revealed that all dimensions of integrated care—in terms of mental health—are manifested in national policy documents in the SADC region. An overview is provided in Table 1.

**Table 1:** Integration strategies manifested in national policy

Integration dimensions	Countries				
	Bots	Mal	Nam	RSA	Zam
*Clinical integration*					
1. Centrality of client needs	x	x	x	x	
2. Case management		x	x	x	x
3. Patient education					
4. Client satisfaction					
5. Continuity	x		x		x
6. Interaction between professional and client	x				
7. Individual multidisciplinary care plan					
8. Information provision to clients	x				
9. Service characteristics		x	x	x	
10. Client participation				x	
11. Population needs	x		x		
12. Self-management	x	x		x	
*Professional integration*					
13. Inter-professional education				x	
14. Shared vision between professionals					
15. Agreements on interdisciplinary collaboration		x	x	x	x
16. Multidisciplinary guidelines and protocols					
17. Inter-professional governance					
18. Interpersonal characteristics					
19. Clinical leadership				x	
20. Environmental awareness					
21. Value creation for the professional					
22. Performance management	x	x	x		x
23. Creating interdependence between professionals	x			x	
*Organisational integration*					
24. Value creation for organisation	x	x	x	x	
25. Inter-organisational governance	x	x	x	x	x
26. Informal managerial network					
27. Interest management	x	x	x	x	x
28. Performance management					
29. Population needs as binding agent	x	x	x	x	x
30. Organisational features	x	x	x	x	x
31. Inter-organisational strategy	x	x	x	x	x
32. Managerial leadership					
33. Learning organisations				x	
34. Location policy	x	x	x	x	x
35. Competency management					
36. Creating interdependence between organisations	x	x	x	x	x
*System integration*					
37. Social value creation	x	x	x	x	x
38. Available resources	x	x	x	x	x
39. Population features	x	x	x	x	x
40. Stakeholder management	x	x	x	x	x
41. Good governance	x	x	x	x	x
42. Environmental climate	x	x	x	x	x
*Functional integration*					
43. Human resource management	x	x	x	x	
44. Information management	x		x	x	x
45. Resource management		x		x	x
46. Support systems and services					
47. Service management	x	x			
48. Regular feedback of performance indicators	x	x	x	x	x
*Normative integration*					
49. Collective attitude	x	x	x	x	x
50. Sense of urgency	x	x	x	x	x
51. Reliable behaviour					
52. Conflict management					
53. Visionary leadership	x	x	x	x	x
54. Shared vision	x	x	x	x	x
55. Quality features of the informal collaboration					
56. Linking cultures	x		x		x
57. Reputation					
58. Transcending domain perceptions					
59. Trust					

### Clinical integration

Several strategies emerged in terms of clinical integration (coordination of person-focused care in a single process across time, place and discipline—see Additional file 1). The *centrality of client needs* was a consideration forwarded in most of the policies, and included elements such as ensuring protection against discrimination and providing sheltered employment for patients suffering from mental illness (Botswana); the provision of “integrated, promotive, preventive, curative and rehabilitative mental health services” (Malawi); and the integration of mental health and social welfare services to ensure the meeting of all client needs (Namibia). In terms of *case management*, special provisions were made for strategies dealing with vulnerable, high-risk groups and their care (Malawi, Namibia, South Africa, Zambia). *Information provision to clients* was not a prominent strategy, though mention was made of the provision of adequate information to clients (Botswana). *Continuity* manifested in several ways, such as the continuity and harmonisation of comprehensive care by different service providers (Botswana), streamlining “fragmented services/programmes/functions” for example linking programmes with known co-morbidity such as maternal health and tuberculosis (Namibia), and strengthening communication and transport between levels of care within referral processes (Zambia). *Interaction between professional and client* received less focus, but there was mention of the importance of well-trained and supported personnel in ensuring positive client outcomes (Botswana). Regarding *service characteristics*, mention was made of the integration of psychological services with general medical services (Malawi, Namibia, South Africa), though not much focus was placed with its integration with social and welfare services. *Client participation* was referred to in terms of engaging with clients in policy development, implementation, and service planning and monitoring (South Africa), while *population needs* was mentioned as a consideration in service development (Botswana, Namibia). Finally, *self*-*management* as an integration strategy was expressed through the establishment of a patient’s charter highlighting taking responsibility for their own health (Botswana) and the promotion of self-help services (South Africa). In general, most policies touched on some clinical integration strategies. However, it should be kept in mind that the policies assessed were broad, macro-level documents, and individual needs received less focus than population needs in terms of integration strategies. Types of integration such as *patient education*, *client satisfaction*, and *individual multidisciplinary care plan* received little focus.

### Professional integration

Selected professional integration (inter-professional partnerships based on shared competencies, roles, responsibilities and accountability—see Additional file 1) emerged from the policy documents assessed. *Inter*-*professional education* did not receive much focus, though mention was made of the training, support and mentoring of staff working in general health settings, as well as the task-shifting of psychosocial work to non-specialist workers supervised and supported by specialists (South Africa). *Agreements on interdisciplinary collaboration* was especially manifested in directives towards multidisciplinary teams (South Africa), the collaboration among mental health workers and general health workers (South Africa, Namibia, Malawi) and among mental health workers and traditional healers (South Africa, Namibia, Zambia). Mention was made of *clinical leadership* in terms of the positioning of mental health specialists providing mentorship and support to non-specialist health workers in the context of task-shifting (South Africa). *Performance management* was manifested in monitoring and evaluating service provision (Malawi), ensuring that mental health personnel are well-trained and committed (Botswana), and implementing a performance management system (Namibia, Zambia). Finally, *creating interdependence between professionals* was detailed by the strengthening of referral systems, linkages and communication among health care workers (Botswana), as well as task-shifting strategies (South Africa). In general, focus on agreements on *interdisciplinary collaboration* and *performance management* received particular focus. Further, South African policies placed more value on professional integration strategies than its neighbouring states.

### Organisational integration

Organisational integration (inter-organisational relationships, including common governance mechanisms—see Additional file 1) emerged intermittently. *Value creation for organisation* was manifested in the out-contracting of services to NGOs and private organisations (Botswana, Malawi, Namibia, South Africa). Strategies such as aligning programmes among service providers (Botswana), ensuring the participation of private organisations, civil society, traditional healers, and international agencies in service delivery, and a collaborative and referral strategy among the three tiers of health care provision (Botswana, Malawi, Namibia, South Africa, Zambia) were all suggestive of *inter*-*organisational governance,interest management, organisational features, inter*-*organisational strategy,* and *creating interdependence between organisations*. *Population needs* were considered in a description of national health statuses and burden of disease, as well as socio-economic aspects such as poverty (Botswana, Malawi, Namibia, South Africa, Zambia). *Learning organisations*—the idea that organisations should develop and maintain a culture of constant learning by its members—is manifested in directives such as the training in mental health care of non-health related public sector workers and civil society partners through in-service training (South Africa). In terms of *location policy*, not much was mentioned in terms of collaborative initiative among government and non-government organisations sharing facility space, although much attention was paid to government facility decentralisation, the providing of mental health care in PHC clinics, districts and regional hospitals (Botswana, Malawi, Namibia, South Africa, Zambia). Organisational integration strategies are key mechanisms which lead to integrated mental health systems. In the policies assessed, several strategies emerged generally uniformly across different countries. It has to be kept in mind that “organisations” in the country policies refers to both state-funded and non-state health facilities and the relations among them. This is opposed to its meaning in other contexts, for instance in some West European countries where mental health care is provided by independent organisations.

### System integration

System integration (horizontal and vertical integration based on a coherent set of rules and policies between care providers and external stakeholders—see Additional file 1) was widely highlighted in the policies assessed. All country policies acknowledged the importance and value of collaboration in service design and provision, in line with *social value creation*. *Available resources* are manifested in acknowledgements of limited resources, and the need to optimise these resources through appropriate cross-subsidisation and private–public service mix (Malawi, Namibia, South Africa, Zambia); simply put, “The assumption that all health care should be provided by government is, in many countries, unrealistic—the necessary resources simply do not exist.” (Botswana). In terms of *population features*, the regional burden of communicable as well as non-communicable diseases was acknowledged, with HIV especially highlighted; social determinants of health such as poverty and lack of access to health services were also underlined (Botswana, Malawi, Namibia, South Africa, Zambia). *Stakeholder management* and *good governance* were highlighted under directives to engage with stakeholders such as private practitioners, traditional healers, NGOs, and faith-based organisations (Botswana, Malawi, Namibia, South Africa, Zambia). *Environmental climate* was manifested in the particular pluralistic health system configurations of the countries assessed, allowing collaboration among government and non-government service providers; such collaborative activities as well as decentralisation-type integration were central features of the policies. It is not that surprising that system integration received a strong focus among the country policies, given the macro scope of system integration dimensions.

### Functional integration

Several functional integration strategies (key support functions and activities structured around the primary process of service delivery to coordinate and support accountability and decision-making—see Additional file 1) emerged. *Human resource management* was highlighted in terms of directives such as a multi-stakeholder human resource steering committee that is intended to provide strategic oversight related to human resources planning activities (Botswana); agreements with NGOs to train health care workers (Malawi); and mental health training for all health care workers, government as well as non-government (Namibia, South Africa). *Information management* was expressed in directives that promote the standardised collection of data from all health service providers (Botswana, Namibia, South Africa, Zambia). *Regular feedback of performance indicators* was promoted in terms of disseminating monitoring information to stakeholders (Botswana, Malawi, Namibia, South Africa, Zambia). *Resource management* was mentioned in the form of public–private partnerships in constructing and upgrading health facilities (Malawi, Zambia) and the use of community-based resources (South Africa). *Service management* was highlighted in terms of a directive ensuring 24-hour access to mental health services (Botswana, South Africa). While all countries included functional dimensions in their approaches to integrated mental health care, some elements were lacking—most notably, *support systems and services*, and *service management*. Again, these exclusions could be due to the micro scope of the particular integration strategies, as national policy documents often do not directly speak to the operational level.

### Normative integration

The normative dimensions of integrated care (the development and maintenance of a common frame of reference between organisations, professional groups and individuals—see Additional file 1) were more difficult to assess in the policies under scrutiny, principally due to the inherent need for more wide-ranging empirical investigations. This is especially evident in aspects such as *reliable behaviour*, *conflict management*, *quality features of the informal collaboration*, and *trust*. Nevertheless, the aim was to explore strategies that underscore and support normative integration. In this way, *collective attitude* and *sense of urgency* were suggested by aspects such as promoting relationships with and coordinating multiple stakeholders in providing mental health services; the state is often positioned as custodian of health care, but clearly needs input from all service providers within collaborative relationships (Botswana, Malawi, Namibia, South Africa, Zambia). *Linking cultures* was evidenced by directives to harmonise and align health service provision activities across all stakeholders (Botswana, Namibia, Zambia). The mere presence of a national mental health policy that promotes integrated care through collaborative relationships could be taken as an aspect of *visionary leadership* and a *shared vision*, although the common challenge of policy/implementation discordance makes this difficult to assess. Normative integration strategies also involve a relational focus, which makes it difficult to adequately assess the inclusion of dimensions such as collective attitude, reliable behaviour, and quality features of the informal collaboration in national state-driven policies. Finally, the lack of focus on *transcending domain perceptions* and *trust* could undermine the multi-professional aspirations of the mental health system reform strategies pursued by countries in the region.

## Discussion

The purpose of this article was to provide an overview of the scope and focus of integrated mental health care of countries in the SADC region. To this end, the Rainbow Model proved to be a useful tool with which to interrogate relevant strategies that are promoted by national health policy. The findings revealed several strategies related to integrated mental health care, across micro, meso and macro domains. While several strategies were mentioned in the policies, several were also absent. A lack of attention paid to clinical dimensions of integrated mental health care—for instance *client participation*, *information provision to clients*, *individual multidisciplinary care plan*, *client satisfaction*, and *patient education*—were especially worrying given the necessity of integrated care to be patient-focused [39, 56, 58]. Nonetheless, cutting across the six integrated care domains of the Rainbow Model, two broad integration strategies emerged within the policies analysed: one, integrating mental health care into PHC, and two, collaboration in service provision among government and non-government role players, as well as among different government sectors. These broad strokes confirm Flisher and colleagues’ [7] description of Sub-Sahara African mental health care systems.

### PHC integrated mental health care

The integration of mental health into PHC is a well-known strategy in mental health reform processes globally, especially in LMIC settings [28, 72]. At the heart of integrated mental health care are efforts to bring specialist services closer to PHC level service providers [21] which links in well with the dominance of PHC in health systems of the SADC region [73, 74]. The popularity of this approach lies in the parallels between the goals of integrated care and PHC, namely, increasing access to and equity and quality in health care services, as well as reductions in the costs associated with hospital-based health care. Additionally, by moving mental health care from specialist institutions to PHC clinics (i.e. closer to the community), the assumption is that community stigma towards mental illness will be ameliorated [21, 75]. The importance of integrated primary mental health care is underlined by recent developments in South Africa, where mental health is being integrated with other chronic disease programmes on facility, community and population level—in a similar fashion to an integrated chronic disease management model [76]. Such initiatives will no doubt be very much contingent on integration outcomes across the Rainbow Model spectrum.

In the policies assessed, integrating mental health into PHC was a central feature of reform strategies although these strategies were by no means uniform. For instance, some policies described this kind of integration simply as “integration of mental health into PHC”, which means that efforts will be made to provide mental health at PHC facilities. Nevertheless—in line with the lack of professional integration dimensions highlighted in the policies—it remains unclear whether this means that mental health services will be provided by dedicated mental health professionals, whether it will be integrated with other PHC programmes such as maternal and child health, or whether all PHC staff will be trained to provided mental health services. The exception in this regard is South Africa, where a task-shifting approach is forwarded which involves the training, mentoring and supervision of lay health workers by specialist mental health practitioners to provide basic mental health services. This approach has been well-described [29, 77–80].

Possible concerns could be raised in terms of integrating mental health into PHC. Some integration aspects lacking in the policies, while arguably not essential, could at least prove to be influential in the implementation of integrated primary mental health care. These include professional integration components such as *shared vision between professionals*, *inter*-*professional governance*, and *value creation for the professional*. Further, *normative integration* components such as *trust*, *transcending domain perceptions*, *reliable behaviour* and *conflict management* were found to be largely lacking. These professional and normative—or soft—dimensions of integrated care are suggested to be especially salient influences in its implementation on PHC level [44]. Ideological and cultural differences among professionals, as well as poor conflict resolution practices, have been suggested to impede inter-professional collaboration. For instance, the diagnosis and effective management of mental illness in PHC settings has been suggested to raise resistance among general health care practitioners [81]. Petersen [82, 83] highlighted the ways in which dominant biomedical discourses in PHC settings impede mental health care provision in South Africa, while Patel and colleagues [84] noted a lack of clarity in objective setting and outlining of the responsibilities of professionals and managers in integrated primary mental health care efforts. By neglecting such strategies, the integration of mental health into PHC might be met by a range of challenges that may otherwise have been leveraged by normative and professional strategy inclusion.

### Inter-service collaboration

The second current of integrated care in the policies was inter-service collaboration. Comprehensive mental health care denotes a range of psychological, medical and social services, which in turn needs to be coordinated and organised within a multifaceted effort [21, 85, 86]. Collaboration among different service providers is an established feature of modern health systems, and a key strategy in continuity of care and effective resource utilisation [87]. More specifically, successful integration efforts are strongly related to the extent of public, private and voluntary sector collaboration [64]. The presence of such collaboration in health policy is partially due to the assumption that the struggle to effectively respond to rising demands for health services is in part due to a lack of partnership between the state, and private and voluntary sectors [88]. While the private sector—due to its for-profit nature and financial and working conditions incentives—often have superior human and other resources, the non-profit sector have been suggested to increase human rights of people with mental illness within reforms towards integrated mental health care [89]. The policies under focus frequently emphasised the need for collaboration among stakeholders; these were mostly between state-funded health service providers, and between state-funded health service providers and private and non-profit providers. Additionally, collaboration with traditional healers was frequently mentioned, a key consideration in mental health reforms in the SADC region [90–93].

Some normative dimensions of integrated care related to inter-organisational collaboration were not well articulated. Examples such as *linking cultures* and *transcending domain perceptions* have been underlined as important mechanisms influencing successful collaboration within integrated care [64]. An especially salient aspect of inter-organisation collaboration in integrated mental health care is *trust*—which, although it has been increasingly perceived as essential by national governments [94], was not well emphasised. Trust has often been suggested to be a precondition for successful inter-organisational collaboration [94, 95], a lack of which has been described in terms of hostility, mistrust and fighting [96]. Similar to inter-organisational relations, trust has also been suggested to be an essential aspect of inter-professional collaboration [97], elevating its importance in the aforementioned integrated primary mental health care strategies. Finally, the importance of trust is intimately tied to power relations [64, 96], an element to some extent absent in the Rainbow Model. A paucity of knowledge remains in terms of the power dynamics at play in integrated care initiatives—along with the governance of the relations within which they play out. For instance, efforts towards the shifting of mental health services towards PHC clinics might well result in a less central role for hospitals in service delivery, although hospitals will have a substantial power advantage over their PHC partners in the service network [98].

### Integrated mental health care, the Rainbow Model and regional governance

Although buoyed by its robust development and comprehensiveness, the Rainbow Model failed to identify additional macro-level elements of integrated care. Important aspects such as national economic and legal frameworks that are crucial in supporting the implementation of integrated care models can therefore be neglected in an analysis like the present. On regional level, sustained progress in global mental health requires close engagement with, among others, governments [99]. Regional strategies are important influences in priority setting for mental health [100]. The WHO Africa Regional Strategy for Mental Health 2000–2010 [101] called for countries to adopt both the integration of mental health into general and primary health care, as well as to increase collaboration among relevant stakeholders. The SADC Protocol on Health [62] calls for collaboration and harmonisation of health system activities among its member countries, as well as mutual support and assistance in mental health care, including its integration into PHC systems. The advantages and stabilising effects of such a strategy were illustrated in regionally harmonising mental health policies, legislation, information systems and general structures [102]. Among the policies analysed in this article, Botswana, South Africa and Zambia recognised the significance of supra-national approaches to health and health care, for instance calling for inter-country collaboration in developing human resources for health, and calling for good regional health governance. SADC—specifically the SADC Secretariat—could potentially play a significant role in strengthening integrated mental health care development. Possibilities include the support of regional civil society and the training and retention of mental health professionals [60, 61, 103]. Evidence-based interventions such as collaborative stepped care, task-sharing and alternative approaches to human resources for health development [30]—key aspects of integrated care—could be supported from a regional governance level. Regional support could also be instrumental in supporting contemporary collaborative mental health initiatives on population, community and neighbourhood levels recently outlined [104].

The neglect of integrated care related to decentralisation processes—a key aspect of LMIC health system reform—is telling. The absence of such strategies could be ascribed to the acknowledged lack of macro-policy expert input in the development of the Rainbow Model [44]. Also, an argument could be made for the differences in health system configurations in the content of the Rainbow Model. In the countries under scrutiny health services are offered in a pluralistic, free market type systems. In contrast, the bulk of integrated care research has been based in Beveridge (state-provided health care largely financed by public taxation, e.g. UK, Canada) and Bismarck (state coordination and regulation of health care instead of provision, e.g. Belgium, The Netherlands) models of health systems. Health system configurations have been shown to be influential in the provision of integrated care and how it its related policies are implemented, as they significantly influence the positions and power of the state and other stakeholders, and the ways in which health system processes such as integrated mental health care are governed [105, 106]. Ultimately it can be argued that the Rainbow Model leans towards individualistic (as opposed to collective) values, in line with the knowledge base chiefly derived from European and North American contexts. This argument is strengthened by the frequent emphasis in the policies under discussion of the input, consideration of and collaboration with local communities in the provision of integrated mental health care—an aspect not picked up by the model.

The study has several limitations. Several policy documents were omitted in the analysis due to time constraints, which include national policies on human resources for health and PHC. The ommitance of pertinent social policies such as those dealing with crime and education is a limitation, and opens up an area for future exploration. Contradictions and coherence across the national policy spectrum will no doubt hold consequences for integrated mental health care. The vagueness with which strategies were described in the policies necessitated that subjective assumptions were made in categorising these strategies under relevant forms of integrated care. Nevertheless, it should be stressed that the purpose of the present article was not to measure integrated care as such, but rather to clarify its strategic meaning. Additionally, the discrepancy between policy content and implementation is well-known, and the integration strategies discussed do by no means reflect real-life integrated mental health care in the countries assessed. SADC countries without mental health policies were omitted under the assumption that the lack of explicit policy documents equates a lack of integrated mental health care, which might not necessarily be the case. It is important to keep in mind that the present analysis took place on the strategic, rather than operational, level.

## Conclusion

Within the contexts of global reform initiatives, mental health care systems are changing. Integrated mental health care is an established feature of these restructurings, and fill a particularly important place in LMIC reforms. It promises to move us closer towards long-held aspirations for quality, equitable and accessible mental health care for those traditionally situated on the peripheries of society. However, this potential is significantly contingent on political will, both on national and supra-national levels. While political resolve is captured in policy, the details are more than just discourse; it is of crucial importance that we are clear about the scope and focus of integrated mental health care in order to better plan and facilitate implementation efforts.

The findings build on recent attempts to clarify integrated mental health care by investigating its meaning both on national and regional levels, significantly drawing from a robust model and applying it to LMIC contexts. Much progress has been made during past decades towards the provision of integrated mental health care under the guise of established models of care. Nevertheless, the present findings highlight the absence of important supportive integrated care strategies which could prove to be influential in the translation of intentions into reality.

The Rainbow Model proved to be a useful tool with which to interrogate a complex health system strategy. Despite its minimal drawbacks, the model lays a strong foundation for prospective empirical research. In this respect, future studies should be mindful of the multi-layered nature of integrated mental health care, which paves the way for empirical fieldwork that explores the finer nuances of the ways in which integrated mental health care unfolds—particularly in LMICs. Such knowledge will no doubt prove to be useful amendments to frameworks such as the Rainbow Model. Further, given the global nature of health system dynamics, more research is needed on the ways in which regional governance could contribute to mental health system reform. Ultimately, theory-led insights on integrated care are decisive to its deployment success and in shaping better mental health systems.

## Additional file

10.1186/s13033-016-0081-7 The Rainbow Model of integrated care. Table outlining the Rainbow Model of integrated care.

## Abbreviations

DALYs: disability-adjusted life years
LMICs: low-to-middle income countries
MDGs: millennium development goals
PHC: primary health care
SADC: South African Development Community
SDGs: sustainable development goals
WHO: World Health Organization

## Footnotes

1 Current member countries: Angola, Botswana, Democratic Republic of Congo, Lesotho, Madagascar, Malawi, Mauritius, Mozambique, Namibia, Seychelles, South Africa, Swaziland, United Republic of Tanzania, Zambia, Zimbabwe.
