# Supplementary material for: Health policy and integrated mental health care in the SADC region: strategic clarification using the Rainbow Model
Source: Int J Ment Health Syst. 2016 Jul 22;10:49. doi: 10.1186/s13033-016-0081-7 (PMC4957874; doi:10.1186/s13033-016-0081-7)
Supplement: Supplementary file 1 — 10.1186/s13033-016-0081-7 The Rainbow Model of integrated care. Table outlining the Rainbow Model of integrated care. [file 13033_2016_81_MOESM1_ESM.docx]

**Additional File 1**

*The Rainbow Model of Integrated Care (Valentijn et al. 2015)*

| **Micro Level** | **Clinical Integration**  The coordination of person-focused care in a single process across time, place and discipline. | |
| --- | --- | --- |
|  | 1. Centrality of client needs | *The principle of care is to address the needs of clients in terms of medical, psychological and social aspects of health.* |
|  | 2. Case management | *Coordination of care for clients’ with a high risk profile (e.g. identifying risks, developing policies and guidance).* |
|  | 3. Patient education | *Education for clients is focused on medical, psychological and social aspects of health.* |
|  | 4. Client satisfaction | *User satisfaction of the individual client is central to the organisation of care.* |
|  | 5. Continuity | *The organisation of care aims to provide fluid care delivery for an individual client.* |
|  | 6. Interaction between professional and client | *Attitude and behavioural characteristics between professional and client regarding all health needs of the client.* |
|  | 7. Individual multidisciplinary care plan | *Implementation of a multidisciplinary care plan at the individual client level.* |
|  | 8. Information provision to clients | *Provide unambiguous and understandable information at the individual client level.* |
|  | 9. Service characteristics | *Provision of services is focused on medical, psychological and social aspects of health.* |
|  | 10. Client participation | *Clients are (pro)actively involved in the design, organisation and provision of care at the operational level.* |
|  | 11. Population needs | *The interdisciplinary approach is consistent with the dominant needs of the population.* |
|  | 12. Self-management | *Tailor-made support of self-management at the individual client level.* |
| **Meso Level** | **Professional Integration**  Inter-professional partnerships based on shared competencies, roles, responsibilities and accountability to deliver a comprehensive continuum of care to a defined population. | |
|  | 13. Inter-professional education | *Inter-professional education for professionals focused on interdisciplinary collaboration.* |
|  | 14. Shared vision between professionals | *A shared vision between professionals focused on the content of care.* |
|  | 15. Agreements on interdisciplinary collaboration | *Agreements on the establishment of interdisciplinary cooperation at the operational level.* |
|  | 16. Multidisciplinary guidelines and protocols | *Multidisciplinary guidelines and protocols are implemented in coherence with the operational level.* |
|  | 17. Inter-professional governance | *Inter-professional governance is focused on openness, integrity and accountability between professionals at the operational level (e.g. joint accountability, appeal on pursued policies and responsibilities).* |
|  | 18. Interpersonal characteristics | *Interpersonal characteristics of the professionals involved in the partnership (e.g. trust, equality, respect, values).* |
|  | 19. Clinical leadership | *Accepted leadership with power and influence at the operational level (e.g. professional status characteristics such as reputation, specialization, position and seniority).* |
|  | 20. Environmental awareness | *Environmental awareness of professionals with regard to economic, social and political developments.* |
|  | 21. Value creation for the professional | *Value is added for the individual professional through interdisciplinary collaboration.* |
|  | 22. Performance management | *Performance management at the operational level is focused on improving health outcomes for the individual client and the population.* |
|  | 23. Creating interdependence between professionals | *Creating mutual interdependencies between professionals regarding interdisciplinary collaboration.* |
| **Meso Level** | **Organisational integration**  Inter-organisational relationships (e.g. contracting, strategic alliances, knowledge networks, mergers), including common governance mechanisms, to deliver comprehensive services to a defined population. | |
|  | 24. Value creation for organisation | *Value is added through the collaboration of each involved organisation.* |
|  | 25. Inter-organisational governance | *Inter-organisational governance is focused on openness, integrity and accountability between organisations at the strategic level (e.g. joint responsibilities, strategy and policy).* |
|  | 26. Informal managerial network | *Informal network of managers within the collaboration.* |
|  | 27. Interest management | *A climate that attempts to bridge the various interests (e.g. social, organisational and personal) at the operational, tactical and strategic level.* |
|  | 28. Performance management | *Collective elaborated performance management between organisations within the collaboration.* |
|  | 29. Population needs as binding agent | *The needs of the population are central in the collective policy of the various organisations in the collaboration.* |
|  | 30. Organisational features | *Organisational features of inter-organisational collaboration (e.g. legal structure, number of organisations, profit vs. non-profit).* |
|  | 31. Inter-organisational strategy | *A collective elaborated strategy exists between the organisations within the collaboration.* |
|  | 32. Managerial leadership | *Leadership with power and influence at a strategic level (e.g. reputation, seniority and formal position).* |
|  | 33. Learning organisations | *Collective learning power between the organisations within the collaboration (e.g. joint research and development programs).* |
|  | 34. Location policy | *A collective location policy between the organisations within the collaboration (e.g. coordinated housing and facilities).* |
|  | 35. Competency management | *Collectively utilize and select competencies of professionals and staff to the greatest possible extent for the objectives of the collaboration.* |
|  | 36. Creating interdependence between organisations | *The organisation of the collaboration aims to create mutual interdependencies between organisations (e.g. multiyear rental agreement).* |
| **Macro Level** | **System integration**  A horizontal and vertical integrated system, based on a coherent set of (informal and formal) rules and policies between care providers and external stakeholders for the benefit of people and populations. | |
|  | 37. Social value creation | *Value is added through the collaboration of social objectives and interests.* |
|  | 38. Available resources | *Available resources in the environment of the collaboration (e.g. usable buildings, (over)capacity, professionals and funding streams).* |
|  | 39. Population features | *Health determinants of the population in the environment of the partnership (e.g. population composition and use of care).* |
|  | 40. Stakeholder management | *Engagement of various stakeholders (e.g. municipality, patient organisations and health insurance companies).* |
|  | 41. Good governance | *Creating trust towards external stakeholders (e.g. municipality and health insurance companies) based on working method, reputation, management, control and/or supervision.* |
|  | 42. Environmental climate | *Political, economic and social climate within the environment of the collaboration (e.g. market characteristics, regulatory framework, competition).* |
| **Macro, Meso, Micro Levels** | **Functional integration**  Key support functions and activities (i.e. financial, management and information systems) structured around the primary process of service delivery to coordinate and support accountability and decision-making between organisations and professionals in order to add overall value to the system. | |
|  | 43. Human resource management | *Aligned Human Resource Management within the collaboration (e.g. joint staffing and personnel).* |
|  | 44. Information management | *Aligned information management systems accessible at an operational, tactical and strategic level (e.g. monitoring and benchmarking systems).* |
|  | 45. Resource management | *Coherent use of resources (e.g. collective real estate and funding).* |
|  | 46. Support systems and services | *Aligned support systems and services at the operational level (e.g. facility management and secretarial support).* |
|  | 47. Service management | *Aligned service management for the client (e.g. collective telephone numbers, counter assistance and 24-hour access).* |
|  | 48. Regular feedback of performance indicators | *Regular feedback of performance indicators for professionals at the operational level to enable them to improve their performance.* |
| **Macro, Meso, Micro Levels** | **Normative Integration**  The development and maintenance of a common frame of reference (i.e. shared mission, vision, values and culture) between organisations, professional groups and individuals. | |
|  | 49. Collective attitude | *Collective attitude within the collaboration towards open communication, sincerity and respect at operational, tactical and strategic levels.* |
|  | 50. Sense of urgency | *Awareness regarding the need and purpose to collaborate at the operational, tactical and strategic levels.* |
|  | 51. Reliable behaviour | *The extent to which the agreements and promises within the collaboration are fulfilled at operational, tactical and strategic levels.* |
|  | 52. Conflict management | *The ability to effectively manage interpersonal conflicts within the collaboration.* |
|  | 53. Visionary leadership. | *Leadership based on a personal vision that inspires and mobilizes people* |
|  | 54. Shared vision | *A collectively shared long-term vision within the collaboration at the operational, tactical and strategic levels.* |
|  | 55. Quality features of the informal collaboration | *Effectiveness and efficiency of the informal collaboration at the operational, tactical and strategic levels (e.g. group dynamics and attention to the undercurrent).* |
|  | 56. Linking cultures | *Linking cultures (e.g. values and norms) with different ideological values within the collaboration at the operational, tactical and strategic levels.* |
|  | 57. Reputation | *Individual reputation of those people involved in the collaboration.* |
|  | 58. Transcending domain perceptions | *The ability to transcend one’s own professional domain within the collaboration at the operational, tactical and strategic levels.* |
|  | 59. Trust | *The extent to which those involved in the collaboration at operational, tactical and strategic levels trust each other.* |
